# Exploring therapeutic mechanism of Fuzhenghefuzhiyang Formula in psoriasis: inflammation and metabolism regulation

**DOI:** 10.3389/fimmu.2025.1690070

**Published:** 2025-11-26

**Authors:** Bin Tang, Chen Zhang, Yujie Yang, Haixin Zhong, Kexin Yang, Hao Deng, Jingjie Yu, Yonggen Chen, Xiangliang Deng, Chuanjian Lu, Haiming Chen

**Affiliations:** 1 State Key Laboratory of Dampness Syndrome of Chinese Medicine, The Second Affiliated Hospital of Guangzhou University of Chinese Medicine (Guangdong Provincial Hospital of Chinese Medicine), Guangzhou, China; 2 State Key Laboratory of Traditional Chinese Medicine Syndrome, The Second Affiliated Hospital of Guangzhou University of Chinese Medicine, Guangzhou, China; 3 Guangdong-Hong Kong-Macau Joint Lab on Chinese Medicine and Immune Disease Research, Guangzhou University of Chinese Medicine, Guangzhou, China; 4 Department of Clinical Pharmacy, Guangzhou First People’s Hospital, Guangzhou, China; 5 School of Traditional Chinese Medicine, Guangdong Pharmaceutical University, Yunfu, China

**Keywords:** psoriasis, FZHFZY, transcriptomics, metabolomics, integrated analysis, NLRP3

## Abstract

**Background:**

Psoriasis is a long-term, immune-mediated inflammatory disorder of the skin, affecting about 2-3% of the global population with rising prevalence rates. Clinical practice has demonstrated that Fuzhenghefuzhiyang formula (FZHFZY), a topical medication applied Chinese herbal formula developed by Prof. Lu Chuanjian, exhibits significant efficacy in alleviating psoriasis symptoms. Nevertheless, its mechanism of anti-psoriasis remains unclear.

**Purpose:**

The purpose of this study is to decode the molecular mechanisms by which FZHFZY produces its therapeutic effects on psoriasis using an integrated multi-omics approach.

**Methods:**

Employing both cell-based and animal studies, we systematically elucidate the pharmacodynamic substance basis and biological pathways underlying its anti-psoriasis effects by integrating network pharmacology to predict the active ingredient-target network, metabolomics to analyze endogenous metabolite changes, and transcriptome sequencing to dissect gene expression regulation.

**Results:**

FZHFZY effectively alleviated symptoms of skin thickening, erythema, and scaling in psoriatic model mice. Network pharmacology predicted that its therapeutic effect might be related to target pathways associated with inflammation, metabolism, proliferation, pyroptosis. RNA sequencing analysis of skin lesion tissues revealed that FZHFZY modulated the inflammation-related NOD-like receptor (NLR) and IL-17 signaling pathways. Further validation by RT-PCR showed that FZHFZY markedly downregulated the mRNA levels of IL-17 and NLRP3. Metabolomics analysis of the lesion tissues identified 169 differential metabolites, and FZHFZY was found to regulate the catabolism of carbohydrates, purines, and most amino acids. Cross-analysis of the two omics approaches indicated that purine metabolism and ether lipid metabolism were associated with key genes in the NLR signaling pathway identified by MCODE analysis.

**Conclusions:**

This study revealed that FZHFZY can effectively improve lesion conditions in a psoriasis-like mouse model, and its mechanism of action may be related to the association between purine metabolism, lipid metabolism, and key genes in the NLR signaling pathway.

## 1 Introduction

Psoriasis is a persistent immune-mediated dermatosis presenting with erythematous, scaly skin lesions, impacts about 3% of the global population (1, 2). It presents a major challenge in dermatological therapeutics due to its prevalence and the complexity of management. Topical therapy has become a trending subject in the treatment of psoriasis, favored for its patient compliance, minimal systemic side effects, and targeted impact (3). Particularly recommended for mild to moderate forms, topical treatments aim to mitigate hyperkeratosis with tar or salicylic acid, and inhibit keratinocyte hyperproliferation using vitamin D3 analogs. However, despite the efficacy of first-line dermatocorticoids in reducing inflammation, local use of vitamin A acid drugs such as tazarotene may cause adverse reactions such as local skin irritation (redness, itching, burning pain). While the current treatment landscape for psoriasis includes a variety of options, the limitations of conventional therapies, including potential adverse effects and the lack of a lifelong cure, complicate long-term management (1, 3).

In response to these challenges, traditional Chinese medicine formulas (TCMFs) have risen as promising alternatives, increasingly recognized for their favorable effects and absence of post-treatment adverse effects (4, 5). TCMFs offer the potential to complement or even supplant conventional treatments, thereby enhancing therapeutic outcomes. Research into the mechanisms of TCMFs has expanded our understanding of their beneficial effects on psoriasis (6–8), presenting a valuable addition to existing treatments and addressing some of the unmet needs of conventional topical formulations. This exploration not only enriches the therapeutic arsenal but also opens new avenues for managing this chronic condition more effectively.

Based on the clinical experience and insights of Professor Lu Chuanjian, a renowned expert in dermatology with a focus on Chinese herbal medicine, the FZHFZY has been distilled from years of clinical practice. This Chinese herbal formula has shown substantial treatment benefits for psoriasis patients, which is supported by clinical observations and emerging research findings. And it was approved by the Guangdong Medical Products Administration as a hospital preparation for Guangdong Provincial Hospital of Chinese Medicine in 2023 (Drug master file record number: Z20230031000). The clinical efficacy of FZHFZY has been corroborated by retrospective studies, such as those published by Wang Junyue, which highlighted its effectiveness in managing psoriasis symptoms (9). Concurrently, our foundational research has been exploring the underlying mechanisms of this formula. Utilizing advanced analytical techniques like LC-MS in conjunction with various cell lines, including HaCaT cells, RAW264.7 cells, and HUVEC endothelial cells, we have identified key bioactive compounds such as neoisoastilbin, melittoside, and spinosin present in FZHFZY (10). Our latest research employed a novel approach by integrating transcriptomes and metabolomes profiling to elucidate the therapeutic mechanisms of this topical formula. This innovative method allowed for a more comprehensive understanding of how FZHFZY modulated the molecular landscape in the treatment of psoriasis.

Previous immunohistochemistry (IHC) has shown that FZHFZY could inhibit the expression level of the macrophage biomarker F4/80 in psoriatic skin, indicating its potential to modulate the immune response. *In vitro* studies using LPS-induced Raw264.7 macrophages have revealed that FZHFZY can mitigate the production of inflammatory cytokines such as TNF-α and IL-6. Furthermore, our research has demonstrated that FZHFZY suppressed the activation of P38/Erk/NF-κB signaling in these macrophages, which were critical in the inflammatory processes associated with psoriasis (11). Based on these findings, we have also shown that in IMQ-induced mouse model of psoriasis, FZHFZY can modulate epidermal differentiation and attenuate the phosphorylation of the Akt/mTORC1/S6K1 pathway (12). These collective studies significantly contributed to our understanding of the multifaceted mechanisms through which FZHFZY exerted its anti-psoriatic effects.

In the treatment of psoriasis, it is recommended for mild patients to choose local therapies. Drugs for local treatment include hormones and carboplatin, but these may lead to adverse reactions, such as hormone dependence and local irritation. FZHFZY has been commonly used in clinical settings, and extensive clinical data has indicated its effective therapeutic results for psoriasis; however, its underpinning mechanism has not been fully elucidated. The present work used bioinformatics analysis, combined with cross-analysis of transcriptomic and metabolomic data from animal lesion tissues, to find that its mechanism was potentially associated with purine metabolism, lipid metabolism, and key genes in the NLR signaling pathway. Our research provided experimental evidence for the potential combined use of FZHFZY with systemic drugs in the therapy of psoriasis.

## 2 Materials and methods

### 2.1 Reagents and antibodies

Imiquimod cream (IMQ) (24094001, Mingxin Pharmaceutical, China); DMEM was purchased Gibco Laboratorie (Gibco, United States); fetal bovine serum was provided by Gibco Laboratories (Gibco, United States); Antibodies specific for NLRP3 (CST, United States), β-actin (CST, United States) and goat anti-rabbit antibodies were procured from Cell Signaling Technology (CST, United States); TRIzol was sourced from Thermo Fisher Scientific (Thermo Fisher, United States); HiScript^®^III RT SuperMix for qPCR (+gDNA wiper) and ChamQ Universal SYBR qPCR Master Mix were supplied by Vazyme Biotech Co., Ltd. (Vazyme, China).

### 2.2 Network pharmacology

Traditional Chinese Medicine Network Pharmacology Platform (INPUT 2.0, http://cbcb.cdutcm.edu.cn/INPUT/Home/) was applied to search for the medicinal compounds and candidate targets of FZHFZY. As a result, the active components and putative molecular targets of FZHFZY could be achieved, and the duplicate values were removed. The Universal Protein Resource (UniProt) database (https://www.uniprot.org/) was then used for normalization of gene names. The related targets of psoriatic skin lesions were extracted from Gene Expression Omnibus (GEO) database.

The potential target genes corresponding to active ingredients of FZHFZY and the related target genes were submitted to Venny 2.1.0 platform (https://bioinfogp.cnb.csic.es/tools/venny/index.html) to identify the key target genes, and the Venn maps were plotted. Then, the hub target genes were uploaded to the STRING 11.0 database (https://string-db.org/cgi/input.pl) to establish the protein-protein interaction network. The networks were analyzed by the plugin molecular complex detection (MCODE) to obtain cluster. The Kyoto Encyclopedia of Genes and Genomes (KEGG) pathway assessment was conducted.

### 2.3 Clinical ethics

The skin tissue samples from psoriasis patients were sourced from Guangdong Provincial Hospital of Chinese Medicine and authorized by the Institutional Review Board (approval number: BB2025-079-01). Prior to participation, every patient gave written informed consent.

### 2.4 Preparation and LC-ESI-MSn analysis of FZHFZY

The FZHFZY formula was purchased by Guangdong Provincial Hospital of Chinese Medicine. The herbs listed in **Table 1** were soaked in water for 30 min and then boiled for 1 h. After two rounds of boiling, the water extracts were combined and the obtained final concentration through electric heating volatilization concentration was 0.5 g/mL (based on crude drug weight/extraction solution volume).

**Table 1:** The composition and proportion of FZHFZY formula.

Ratio	Crude drug weight	Linnean classification	Chinese name	Source
3	30g	*Cynanchum paniculatum Radix etRhizoma*	Xuchangqing	The dried root or rhizome of *Cynanchum paniculatum (Bunge) Kitagawa*
3	30g	*Dictamni Cortex*	Baixianpi	The dried root cortex of *Dictamnus dasycarpus L.*
3	30g	*Granati Pericarpium*	Shiliupi	The dried peel of *Punica granatum L.*
3	30g	*Rehmanniae radix Preparata*	Shudihuang	The prepared root of *Rehmannia glutinosa Libosch.*
3	30g	*Smilax glabra Rhizoma*	Tufuling	The dried rhizome of *the Smilax Glabra Roxb.*
2	20g	*Angelica sinensis Radix*	Danggui	The dried root of *Angelica sinensis (Oliv.) Diels*
2	20g	*Cnidii Fructus*	Shechuangzi	The dried fruit of *Cnidium monnieri (L.) Cuss.*

The DIONEX ULTIMATE 3000 ultra high-performance liquid chromatography (UHPLC) system linked to a Q Exactive Plus Hybrid was employed for chemical profiling of FZHFZY. Quadrupole Orbitrap Mass Spectrometer (Q-Exactive-Series MS) was purchased from Thermo Fisher Scientific, featuring an integrated heated electrospray ionization source. Prior to analysis, 0.1 mL of FZHFZY extract was mixed with 1 mL of 70% methanol and sonicated for 30 min. The resulting solution was filtered through a 0.22 μm membrane prior to UPLC-MS analysis. Chromatographic separation was achieved using an ACQUITY UPLC hSS T3 column (2.1 × 100 mm, 1.8 μm) with a binary mobile phase system comprising (A) 0.1% formic acid in water and (B) acetonitrile, at a flow rate of 0.3 mL/min. The injection volume was 5 μL The chromatographic separation employed a linear gradient program: initial hold at 10% B for 2 min, increased to 20% B over 3 min (2–5 min), held for 2 min (5–7 min), then ramped to 30% B at 8 min. From 8–11 min, the gradient increased to 40% B, followed by a gradual rise to 45% B (11–13 min) and 52% B (13–15 min). A steeper gradient to 70% B was applied (15–18 min), then to 80% B (18–25 min), before returning to initial conditions (10% B at 25–26 min) for column re-equilibration (26–30 min). The flow rate was maintained at 0.2 mL/min with a column temperature of 35°C. Mass spectrometric detection was conducted using Heated Electrospray Ionization (HESI) in both positive and negative modes. The parameters were as follows: spray voltage 2.5 kV, capillary temperature 350°C, auxiliary gas temperature 350°C and auxiliary gas flow rate 15 psi, sheath gas flow rate 40 psi, sweep gas flow rate 0 psi, collision energy 20, 30, 40 V, and a mass range of m/z 200 to 2000 for data collection.

### 2.5 Animals and treatments

Male BALB/c mice aged 6–8 weeks were provided by the Centre of Laboratory Animals of Southern Medical University (Guangzhou, China), and their weight are 20 ± 2 g, were maintained at 22 ± 2°C, 45-55% humidity, with 12 h light/dark cycles in standard housing.

BALB/c mice were randomly allocated into four experimental groups, 10 per group: control group, the IMQ group, calcipotriol cream (Cal) group and FZHFZY group. The dose of FZHFZY administered to each mouse was the optimal dose determined based on previous research (the concentration of FZHFZY is 0.25 g/mL, 0.2 mL/day) (12). Use the Psoriasis Area and Severity Index (PASI) -like score to assess the disease severity (erythema, scales, and thickening) of IMQ-induced psoriasis mouse models (13). The splenic weight index was calculated as (spleen weight/body weight) × 100% and compared across experimental groups (14). The animal experiment in our study was approved and performed in accordance with the Animal Experimental Ethics Committee of Guangdong Provincial Hospital of Chinese Medicine (approval number: 2023126).

### 2.6 Histological analysis

Skin tissue samples from mice were fixed in 4% paraformaldehyde, paraffin-embedded, and sectioned at 5 μm for standard hematoxylin and eosin (H&E) staining and histopathological assessment. Immunohistochemical staining was performed following antigen retrieval in citrate buffer (pH 6.0). Endogenous peroxidase activity was blocked with 3% hydrogen peroxide. Primary antibodies were incubated overnight at 4°C: anti-Ki67 (Servicebio, Wuhan, China) and anti-CD3 (Abcam, Waltham, MA, USA). Slides were imaged using an Olympus IX51 upright microscope.

### 2.7 Measurement of mRNA via RT-PCR

RT-PCR was performed to determine the mRNA levels of NLRP3, IL-6, TNF-α, IL-17A, and IL-1β. According to the manufacturer’s instructions, total RNA was extracted from the mice skin tissue or cells using the TRIzol reagent. The mRNA expression genes was determined via an ABI 7500 Fast Real-Time PCR System (Thermo Fisher Scientific, USA), the primer was listed in **Table 2**.

**Table 2:** Primers used for RT-PCR analysis.

Primer	Forward
NLRP3	(F)5’-ATTACCCGCCCGAGAAAGG-3’ (R)5’-TCGCAGCAAAGATCCACACAG-3’
IL-1β	(F)5’-GCAACTGTTCCTGAACTCAACT-3’ (R)5’-ATCTTTTGGGGTCCGTCAACT-3’
IL-6	(F)5’-CTGCAAGAGACTTCCATCCAG-3’ (R)5’-AGTGGTATAGACAGGTCTGTTGG-3’
IL-17A	(F) 5’-CAGCAGCGATCATCCCTCAA-3’ (R) 5’-TCAGGGTCTTCATTGCGGTG-3’
TNF-α	(F)5’-ACTGATGAGAGGGAGGCCAT-3’ (R)5’-CCGTGGGTTGGACAGATGAA-3’
NOD2	(F)5’-CCTAGCACTGATGCTGGAGAAG-3’ (R)5’- CGGTAGGTGATGCCATTGTTGG-3’
IL-23	(F)5’-CATGCTAGCCTGGAACGCACAT-3’ (R)5’- ACTGGCTGTTGTCCTTGAGTCC-3’
β-actin	(F)5’-GTGACGTTGACATCCGTAAAGA-3’ (R)5’-GCCGGACTCATCGTACTCC-3’

### 2.8 RNA sequencing analysis

RNA extraction from skin tissue was performed with TRIzol reagent. Total RNA concentration quantified using the ND-2000 (NanoDrop Technologies). The high-quality RNA samples were used to construct sequencing library. RNA purification, reverse transcription, library construction and sequencing were performed at Shanghai Majorbio Bio-Pharm Technology Co., Ltd. Using FastP software to perform a series of quality control steps on the raw sequencing data, and conducting further statistical and quality evaluation on the cleaned data. HiSat2 was used to align the cleaned reads to the reference genome. Then the DESeq2 package (v1.36.0) was employed to identify differentially expressed genes (DEGs) based on the expression matrix. DEGs were identified across groups by applying the filtering criteria P < 0.05 and |log2FC| ≥ 1, and were depicted in volcano plots. In addition, we performed KEGG pathway enrichment analysis using the clusterProfiler package on the intersected DEGs from the whole-transcriptome background. GSEA (version 4.1.0) was employed to analyze genome-wide expression profiles by evaluating the enrichment of predefined gene sets. This approach determined whether functionally related gene groups exhibited coordinated expression changes with statistical significance.

### 2.9 Metabolomics analysis

The mice skin tissue (50 mg) was grinded in centrifuge tube, followed by addition of 400 μL of extraction solution (methanol: water = 4:1, v/v) containing 0.02 mg/mL L-2-chlorophenylalanine. After incubation at -20°C for 30 min, samples were centrifuged at 13,000 × g for 15 minutes (4°C). The resulting supernatant was collected for LC-MS/MS analysis. Quality control (QC) samples were processed identically to analytical samples. LC-MS/MS analysis was performed using a Thermo UHPLC-Q Exactive HF-X system (Majorbio Bio-Pharm Technology Co., Shanghai, China) with an ACQUITY HSS T3 column (100 × 2.1 mm, 1.8 μm; Waters, USA). To achieve efficient separation of metabolites in the samples, different time proportions and proportions of mobile phase B were set for positive ion mode and negative ion mode, respectively, in the gradient separation.

LC/MS raw data were processed using Progenesis QI software (Waters Corporation, USA), generating a 3D data matrix (CSV format) containing sample information, metabolite names, and MS response intensities. Metabolite identification was performed by querying the HMDB, Metlin, and Majorbio databases.

Multivariate analysis was performed using the ropls package (v1.6.2) in R, including perform principal component analysis (PCA) and orthogonal partial least squares discriminant analysis (OPLS-DA) with seven-round cross-validation. Metabolites with VIP > 1 and P < 0.05 were considered significant. Pathway mapping was conducted through KEGG metabolic enrichment analysis (www.genome.jp/kegg).

### 2.10 Integrated analysis

Using MetaboAnalyst 5.0 database (https://metascape.org/gp/index.html), select the ”joint-pathway analysis” module to upload core targets obtained from transcriptomic analysis and core metabolites from metabolomic analysis for integrated molecular pathway enrichment. Meanwhile, a compound-response-enzyme-gene network was constructed by the MetScape plugin.

### 2.11 Cell culture

RAW264.7 cells (Storage Centre of Wuhan University) were maintained in DMEM (Gibco, USA) with 10% FBS and 1% penicillin-streptomycin at 37°C/5% CO_2_. For experiments, cells were stimulated with LPS (1 μg/mL, 6 h) followed by FZHFZY treatment (600 or 900 μg/mL, 18 h).

### 2.12 Cell viability assay (CCK8)

Cell viability was assessed using CCK-8 assay. Cells (5×10_4_ cells/mL) were plated in 96-well plates, treated with FZHFZY (0, 150, 300, 600 and 900 μg/mL) for 24 h, then incubated with CCK-8 reagent for 4 h. Absorbance at 450 nm was measured using a Thermo Scientific microplate reader.

### 2.13 Western blotting

Total proteins were obtained by lysing cells with 1×RIPA Lysis Buffer (Beyotime, China) in ice, and subjected to centrifugation at 15,000 rpm, 4°C for 20 min. The total proteins were measured by 8% SDS-PAGE gels. Primary antibodies (NLRP3 and β-actin) were incubated at 4°C overnight, and followed by incubation with HRP-conjugated goat anti-rabbit secondary antibody at 25°C by 2 h. Then the bands were visualized by Bio-Rad Imaging System (Bio-Rad Biosciences, USA).

### 2.14 Statistical analysis

All data presented as mean ± standard (SD). Statistical evaluation was conducted by one-way analysis of variance (ANOVA) for the comparison of sample means between multiple groups using SPSS 24.0 (SPSS Inc., Chicago, Illinois, USA). A P-value < 0.05 indicated statistical significance.

## 3 Results

### 3.1 Ingredients of FZHFZY

**Figure 1:**
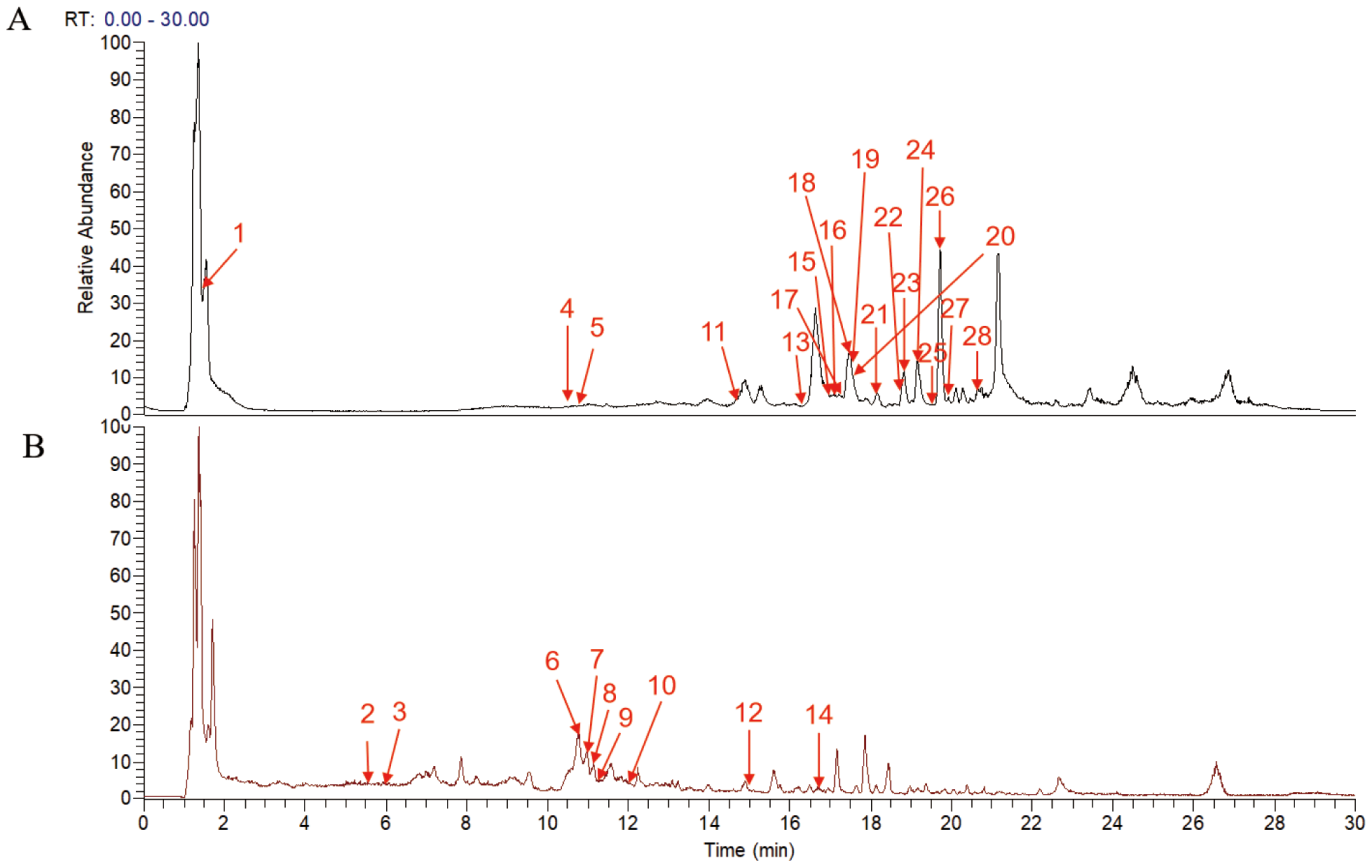
Total ion current chromatogram of the FZHFZY formula in the positive **(A)** and negative **(B)** ionization mode.

UPLC-MS/MS analysis identified 28 major compounds in FZHFZY. The total ion chromatogram (TIC) is shown in **Figure 1**, with MS/MS fragmentation patterns serving as key evidence for identification. Compounds were matched using database comparisons and literature references based on retention times and mass spectral data. Detailed information of these compounds is listed in **Supplementary Table 1**.

### 3.2 Network pharmacology analysis

**Figure 2:**
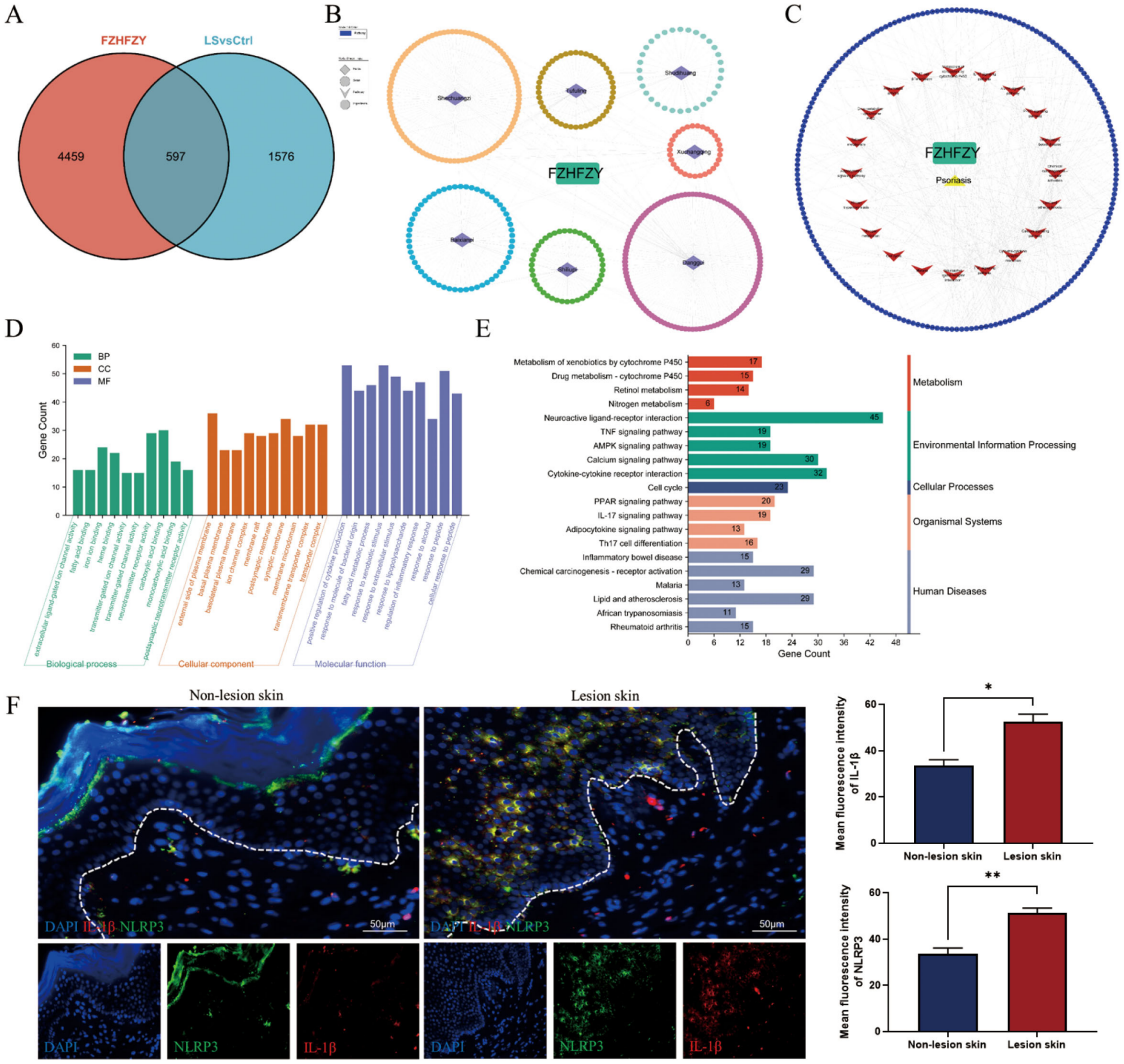
Network pharmacology analysis. **(A)** The Venn map between potential targets of FZHFZY and related target genes of psoriasis. **(B, C)** The PPI network of FZHFZY and psoriasis potential targets. **(D)** The GO enrichment analysis of the biological processes, molecular functions, and cellular components associated with potential common targets. **(E)** The KEGG enrichment analysis of potential common targets. **(F)** Protein expression of NLRP3 and IL-1β in human lesions skin and non-lesions skin (n = 3), *P<0.05, **P<0.01. The data were expressed as mean ± SD.

A total of 5056 active ingredients-related targeted genes of FZHFZY were obtained from the INPUT 2.0 database. The GSE203415, GSE66511 and GSE121212 dataset were selected as the research object, and the dataset were obtained from the GEO database. The inclusion criteria are as follows: (A) Homo sapiens mRNA expression datasets; (B) Datasets including psoriatic lesional skin (LS), non-lesional skin (NL), and healthy control skin (HC), with each group containing at least 10 samples; (C) Experimental type: “high-throughput sequencing expression profile analysis” (**Table 3**). There were 597 targets through Venn 5056 active ingredients-related targeted genes of FZHFZY and the DEGs between the healthy skin and psoriatic lesional skin. The drug-disease intersection targets were 597 (9%) common targets between the DEGs and targeted genes of FZHFZY (**Figure 2A**).

**Table 3:** Information on the datasets.

Dataset ID	Platform	Psoriatic skin lesions	Psoriatic skin non-lesions	Normal	Tissue Type
GSE203415	GPL16791 Illumina HiSeq 2500 (Homo sapiens)	10	10	10	Skin Biopsy
GSE66511	GPL16288 AB 5500xl Genetic Analyzer (Homo sapiens)	12	12	12	Skin Biopsy
GSE121212	GPL16791 Illumina HiSeq 2500 (Homo sapiens)	28	27	38	Skin Biopsy

To elucidate the therapeutic mechanism of FZHFZY in psoriasis, we constructed a compound-target interaction network. The PPI network of FZHFZY was shown in **Figures 2B, C**. GO and KEGG enrichment analysis of the common targets revealed that FZHFZY might modulate the IL-17, TNF, and PPAR signaling pathways. Due to current literature reporting that the onset of psoriasis may be related to pyroptosis, we attempted to explore whether FZHFZY could regulate pyroptosis-related targets (**Figures 2D, E**). Validation through clinical psoriasis patient samples revealed that, compared to non-lesional tissue, the expression levels of IL-1β and NLRP3 were remarkably upregulated in lesional tissue (**Figure 2F**).

### 3.3 FZHFZY attenuated IMQ-induced psoriasis-like skin lesions

**Figure 3:**
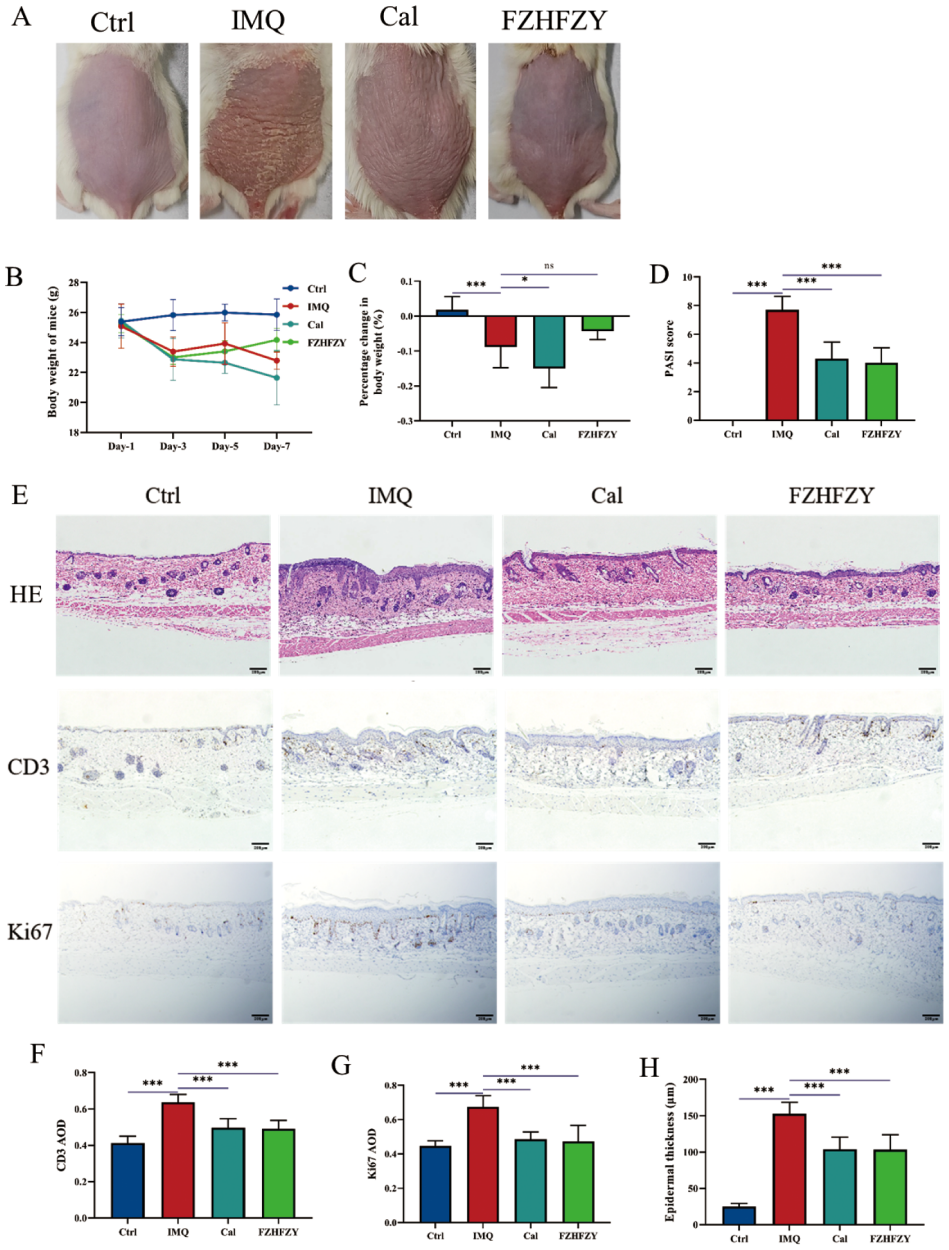
The pharmacological research results of FZHFZY. **(A)** The back skin characteristics of mice in each group (n = 10). **(B)** The weight changes of mice in each group on different days (n = 10). **(C)** The percentage change in body weight of mice in each group (n = 10). **(D)** The PASI scores of mice in each group (n = 10). **(E)** The effect of FZHFZY on the expression levels of H&E, CD3, and Ki67 in IMQ induced psoriasis mice. **(F)** The expression of CD3 of mice in each group (n = 3). **(G)** The expression of Ki67 of mice in each group (n=3). **(H)** The epidermal thickness of mice in each group (n=3). *P<0.05, ***P<0.001. The data were expressed as mean ± SD.

The roles of FZHFZY were investigated in an IMQ-induced psoriasis-like BALB/c mice model. Compared to the dorsal skin of mice in the control group, the dorsal skin of mice in the IMQ group exhibited significant erythema, scaling, and noticeable thickening, resembling human psoriasis-like lesions. The Cal group and FZHFZY group showed reduced scaling and milder erythema compared to the IMQ group, with a marked improvement in the severity of skin lesions (**Figure 3A**). The obtained results are denoted in **Figure 3B**, in parallel to the Ctrl group, the body weight of IMQ-induced psoriasis mice decreased markedly (P < 0.05). The Cal group and IMQ group exhibited a more pronounced decline, while the FZHFZY group showed a milder decrease with a slight rebound in the later stages. In **Figure 3C**, compared to the IMQ group, the Cal group demonstrated the most significant weight loss (P < 0.05), while the FZHFZY group experienced a slightly mitigated reduction. When comparing the FZHFZY group to the Cal group, the weight loss was significantly less (P < 0.05). FZHFZY can modestly alleviate the rate of body weight loss in IMQ-induced psoriasis mice. The PASI scores showed significant improvement (P < 0.01), the treated mice exhibited reductions in erythema, scaling, and infiltration severity, equivalent to the efficacy of the Cal group (**Figure 3D**). Histological assessment of skin lesions was conducted using H&E staining. IHC analysis revealed that the expression levels of CD3 and Ki67 in the FZHFZY group and the Cal group were significantly lower as compared to those in the IMQ group (P < 0.001)(**Figures 3E–H**), the results suggest that the IMQ-induced psoriasis mice exhibited T-cell infiltration and abnormal keratinocyte activity, and treatment with FZHFZY inhibited inflammatory cell infiltration and keratinocyte proliferation.

### 3.4 Transcriptome profiling

**Figure 4:**
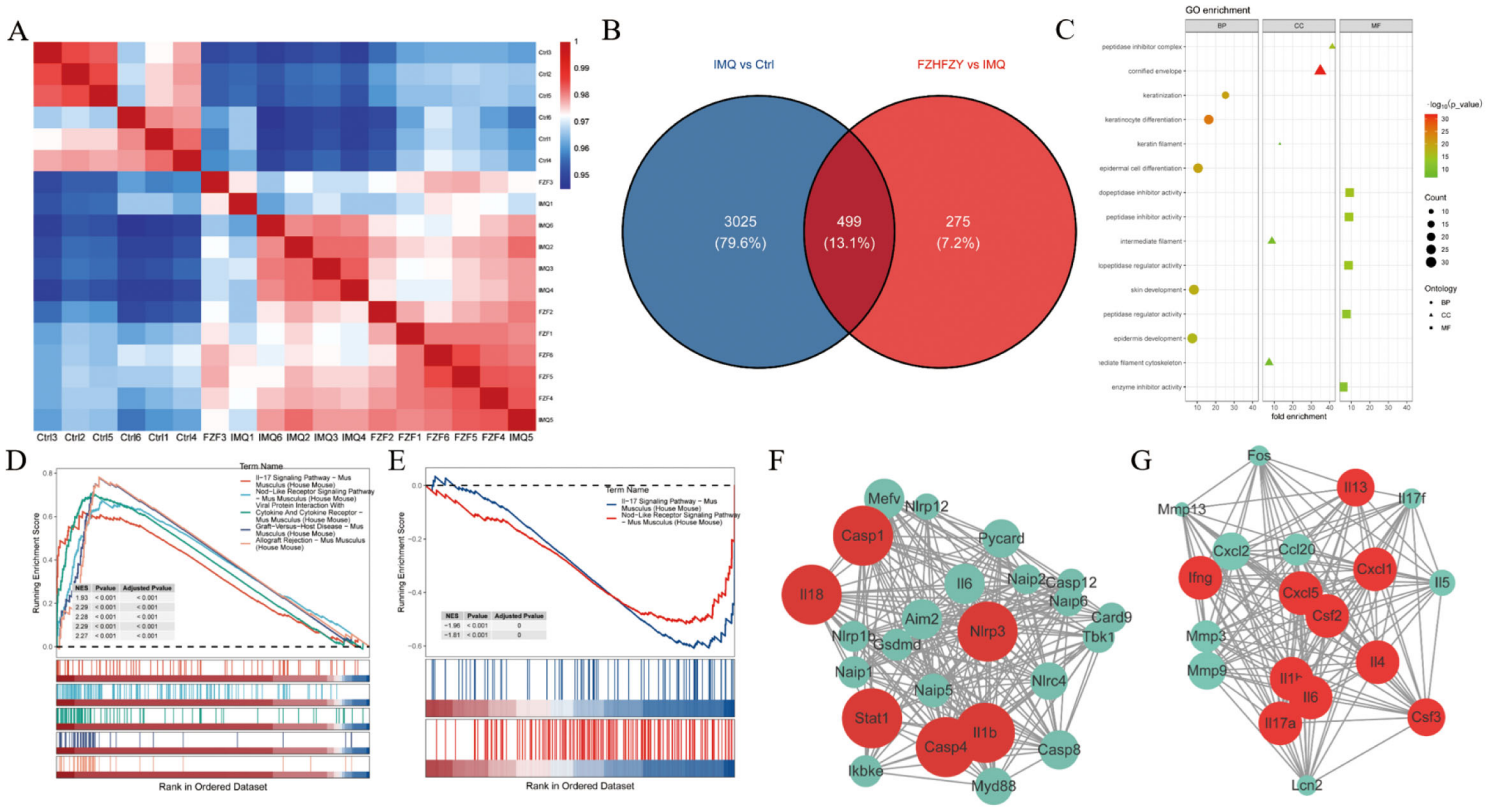
The transcriptome profiling results of FZHFZY. **(A)** The correlation heatmap of samples. **(B)** The Venn map between Ctrl group vs. IMQ group and IMQ group vs. FZHFZY group. **(C)** The GO enrichment analysis with differentially expressed genes. **(D, E)** GSEA enrichment analysis chart. **(F, G)** The PPI network based on MCODE recognition module. n = 6.

The expression matrices of the three groups were normalized. A comprehensive analysis of gene expression data for all samples was conducted, computing pairwise Pearson correlations across samples. It was found that the Pearson correlation coefficients for all samples exceeded 0.9, indicative of strong concordance in transcriptional profiles across samples (**Figure 4A**). DEGs were identified using thresholds of |log 2-Fold Change| ≥ 1 and P value < 0.05 as standard. In contrast to the control group, 3524 DEGs were screened in the IMQ group, including 2353 up-regulated and 1171 down-regulated genes. Compared to the IMQ group, 774 DEGs were identified in the FZHFZY treatment group, comprising 270 up-regulated and 504 down-regulated genes (**Figure 4B**). The volcano map showed the distribution of DEGs (**Supplementary Figure S1A, B**). These DEGs were used for subsequent intersection analysis (**Supplementary Figure S1C**). These DEGs were enriched in cytokine-cytokine receptor interaction, *Staphylococcus aureus* infection, amoebiasis, IL-17 signaling pathway, which indicated that FZHFZY could regulate the inflammation-related pathways. Signal pathways were identified by gene set enrichment analysis (GSEA). In the GO analysis, the GO terms were categorized into three sections: BP (Biological Process), CC (Cellular Component), and MF (Molecular Function), a total of 174 GO terms were identified, comprising 88 biological processes, 17 cellular components, and 69 molecular functions. GO terms with higher enrichment scores were visualized using a bubble chart. Differentially expressed gene enrichment analysis indicated that these genes were primarily involved in key biological processes including epidermis development, epidermal cell differentiation, skin development, and keratinization. These processes were essential for maintaining the physiological structure and function of the skin, encompassing cell proliferation, migration, differentiation, and the eventual formation of mature skin tissue (**Figure 4C**). The top ranked NLR signaling and IL-17 signaling (**Figures 4D, E**) were found to contribute critically to the psoriasis skin lesions and pathways of FZHFZY treatment. The genes of NLR and IL-17 signaling pathway were identified using STRING and further refined via MCODE analysis to pinpoint hub genes (**Figures 4F, G**).

### 3.5 Metabolomics profiling

**Figure 5:**
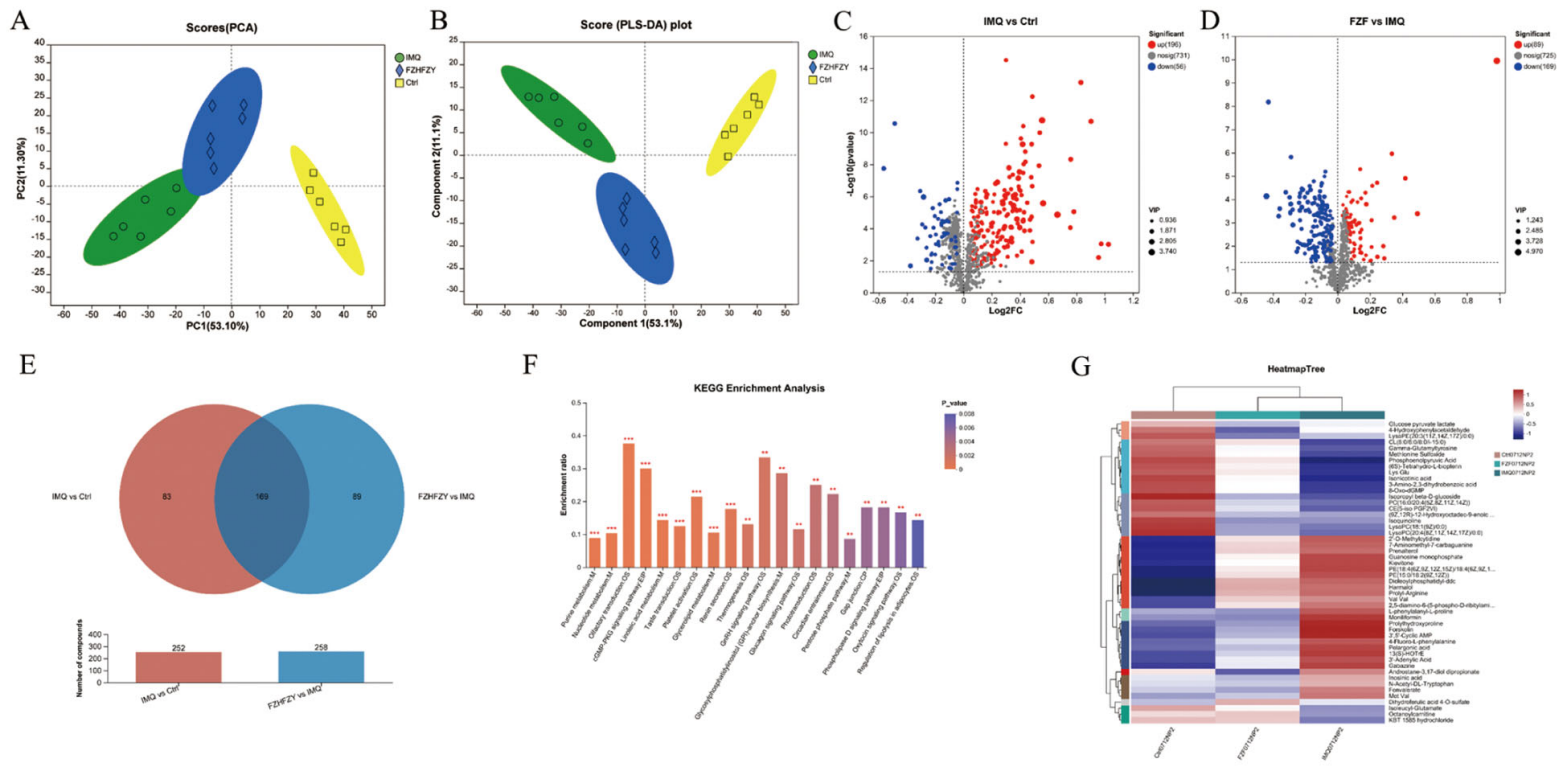
The metabolomics profiling results of FZHFZY. **(A, B)** The results of PCA analysis and PLS-DA analysis. **(C)** The volcano plot of Ctrl group vs. IMQ group. **(D)** The volcano plot of IMQ group vs. FZHFZY group. **(E)** The Venn map between Ctrl group vs. IMQ group and IMQ group vs. FZHFZY group. **(F)** The KEGG enrichment analysis of differential metabolites. **(G)** Metabolite clustering analysis results. n = 6.

QC sample analysis showed consistent instrument performance during the study. The plot of principal component analysis (PCA) of positive ions and negative ions was well separated from three other groups. The cation and anion tables were merged, and Pareto scaling was applied with 95% confidence interval to produce the results of PCA analysis and PLS-DA analysis (**Figures 5A, B**). Metabolites meeting the criteria of VIP > 1 and P < 0.05 were identified as statistically significant, and were visualized as volcano plots (**Figures 5C, D**). The Venn map of the IMQ vs Ctrl group and FZHFZY vs IMQ group revealed 169 significantly different metabolites (**Figure 5E**). The metabolites were enriched in metabolism pathway, such as purine and nucleotide metabolisms (**Figure 5F**). FZHFZY treatment effectively restored most metabolites, and improved the disorder of metabolites in the process of carbohydrate, purine and most amino acid catabolism (**Figure 5G**).

### 3.6 Integrated analysis

**Figure 6:**
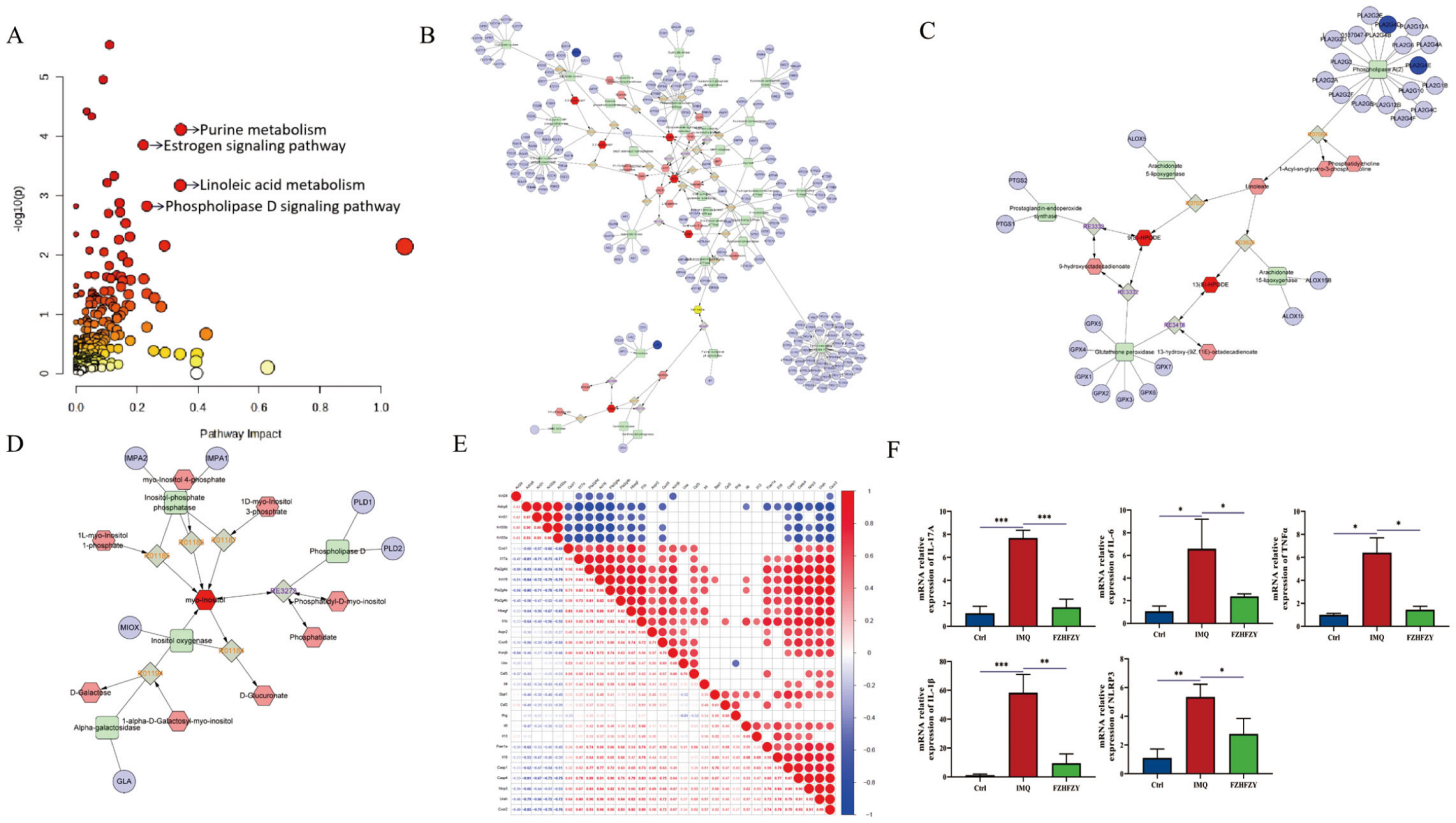
Integrated analysis between differential metabolites and the transcriptome. **(A)** Topology diagram of omics integration. **(B)** Compound response enzyme gene synthesis network of FZHFZY in purine metabolism. **(C)** Compound response enzyme gene synthesis network of FZHFZY in linoleate metabolism. **(D)** Compound response enzyme gene synthesis network of FZHFZY in phosphatidylinositol phosphate metabolism. **(E)** Integrated analysis of key genes and heat map analysis of NLRP3. **(F)** The mRNA expression levels of inflammation-related cytokines IL-17A, IL-6, IL-1β, and TNF-α (n = 3). *P<0.05, **P<0.01, ***P<0.001. The data were expressed as mean ± SD.

The integration of transcriptomic and metabolomic analysis revealed the following pathway results: Purine Metabolism Pathway: compounds: 231, matched compounds: 12, impact: 0.34314. Estrogen Signaling Pathway: compounds: 142, matched compounds: 9, impact: 0.22078. Linoleic Acid Metabolism Pathway: compounds: 78, matched compounds: 6, impact: 0.34146. Phosphatidylinositol-D Signaling Pathway: compounds: 159, matched compounds: 8, impact: 0.23188, and annotated on the pathway map according to the impact magnitude (impact > 0.2, P < 0.05) (**Figure 6A**). To obtain a more comprehensive insight into compound-response-enzyme-gene network of FZHFZY treatment psoriasis, we conducted an integrated analysis of the DEGs identified in RNA-sequencing and differential metabolites (**Figures 6B–D**). The DEGs and altered metabolites were analyzed using the MetScape plugin to construct. It was found that FZHFZY treated psoriatic skin lesion by regulating purine metabolism and linoleic acid metabolism. This study used heatmap analysis to visually demonstrate the relationship between the integrated analysis results and key genes in the NLR and IL-17 signaling. The heat map (**Figure 6E**) analysis showed a significant negative correlation between Adcy8 (adenylate cyclase 8) and NLRP3, with a correlation coefficient of -0.68, suggesting that Adcy8 and NLRP3 might interact with each other in the purine metabolism pathway. The quantitative PCR analysis indicated that FZHFZY effectively down-regulated the transcriptional levels of inflammation-related cytokines IL-17A, IL-6, IL-1β, and TNF-α in skin tissue. These cytokines play a key role in psoriasis pathophysiology, and their overexpression was closely associated with inflammatory response and the formation of skin lesions (**Figure 6F**).

### 3.7 The anti-inflammatory impact of FZHFZY on IMQ-induced psoriasis mouse models

**Figure 7:**
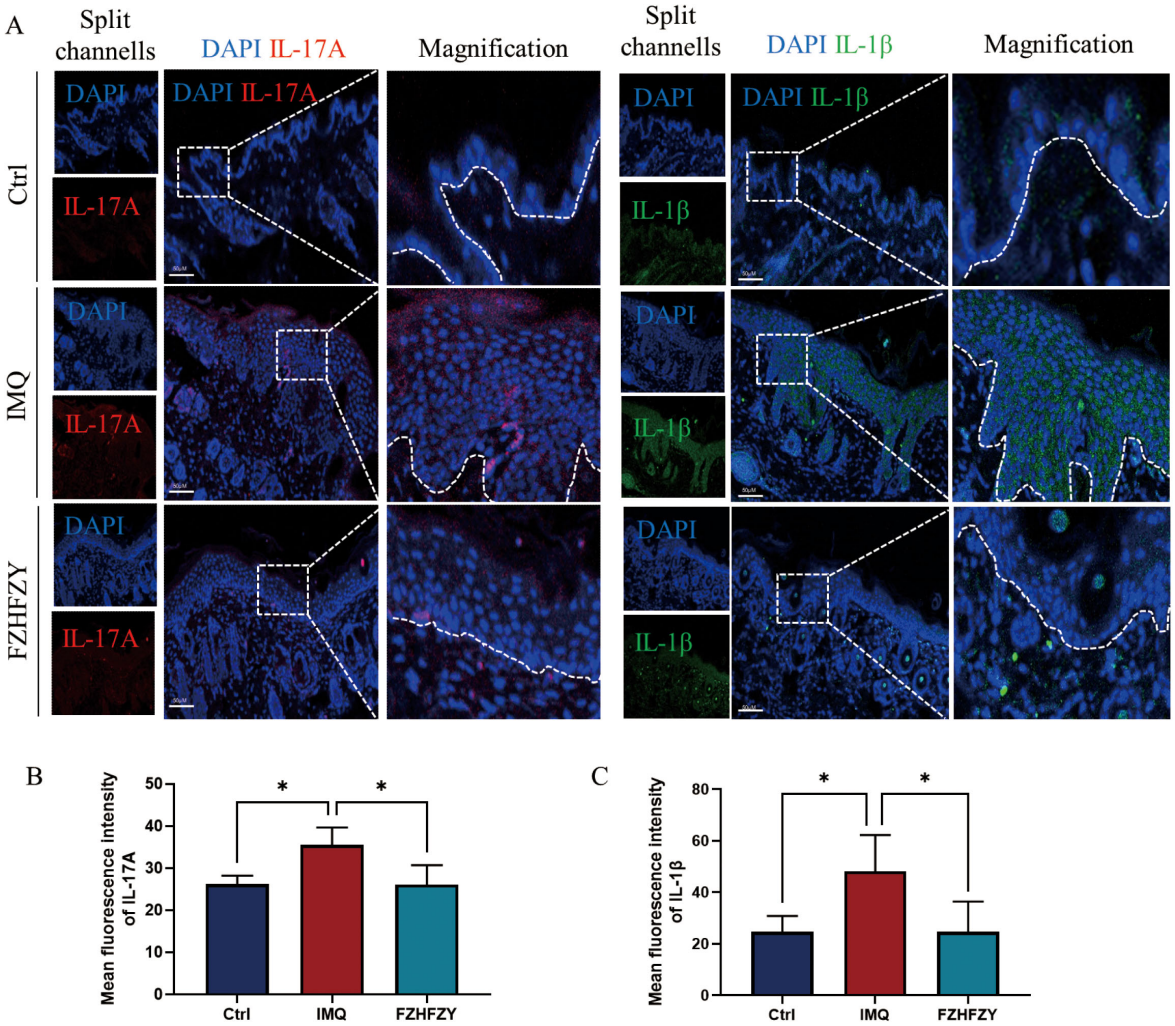
The fluorescence intensity expression of IL-17A and IL-1β. **(A)** The fluorescence intensity expression of FZHFZY on IMQ-induced psoriasis mouse models. **(B)** Mean fluorescence intensity of IL-17A. **(C)** Mean fluorescence intensity of IL-1β. *P<0.05. The data were expressed as mean ± SD, n = 3.

The formula for strengthening the body and relieving itching has shown significant anti-inflammatory activities in the therapy of IMQ-induced psoriasis mouse models. A marked decrease in the fluorescent signal intensity of IL-17A, and IL-1β was observed, indicating that FZHFZY effectively downregulated the protein expression of these pro-inflammatory mediators (**Figures 7A–C**).

### 3.8 The anti-inflammatory impact of FZHFZY on LPS-induced cells

**Figure 8:**
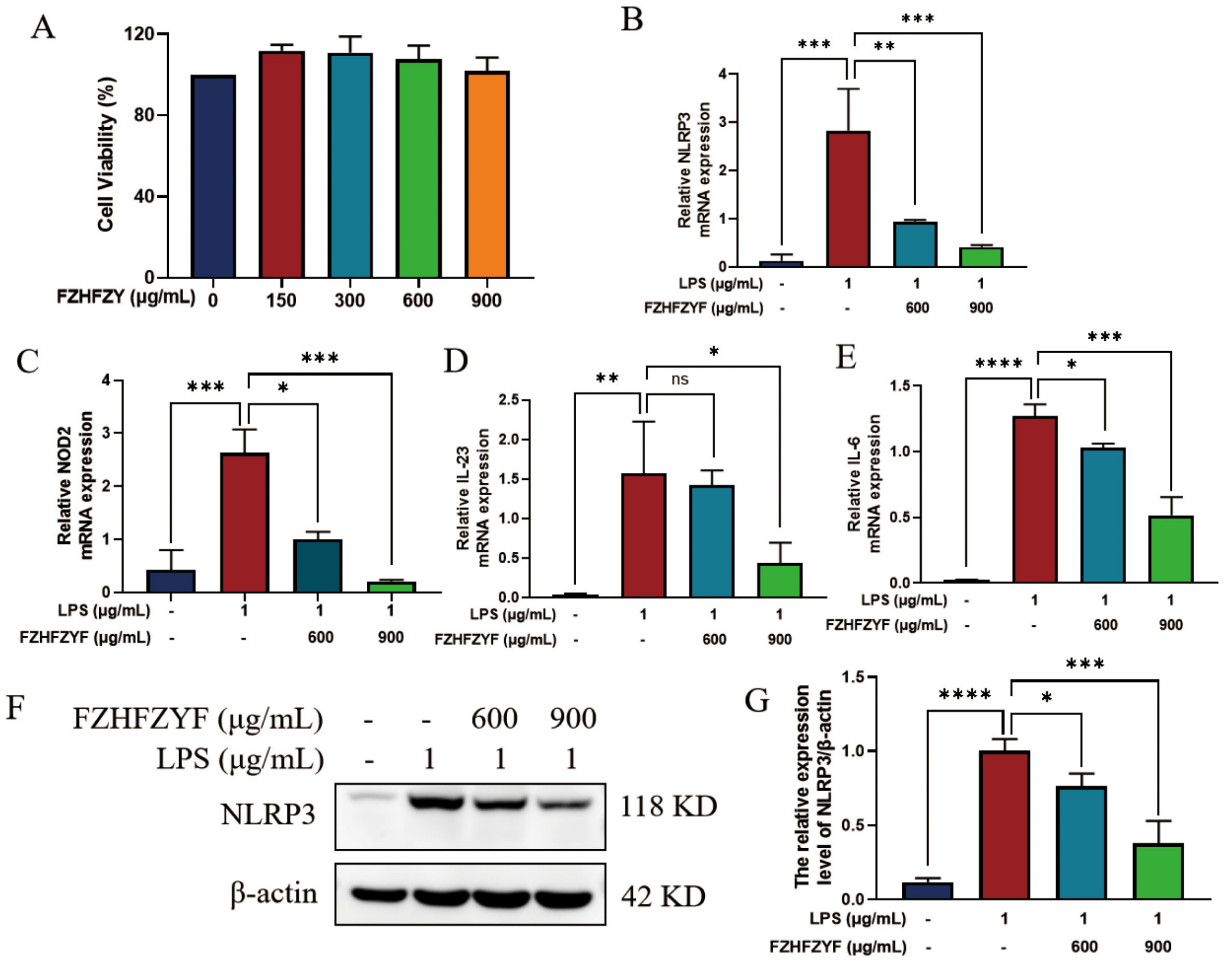
The anti-inflammatory effect of FZHFZY on LPS-induced cells. **(A)** The experimental result of CCK8. **(B–E)** The mRNA expression of NLRP3, NOD2, IL-6 and IL-23. **(F, G)** The protein expression of NLRP3. *P<0.05, **P<0.01, ***P<0.001, ****P<0.0001. The data were expressed as mean ± SD, n = 3.

In the present work, we investigated the effect of FZHFZY on the inflammatory response in an inflammatory cell model induced by LPS. FZHFZY showed no cytotoxicity to RAW264.7 cells at doses of 600 μg/mL and 900 μg/mL (**Figure 8A**). After 24 h of FZHFZY treatment, the expressions of genes including NLRP3, NOD2, IL-6, and IL-23 were significantly downregulated (**Figures 8B–E**). Protein level analysis also confirmed a decreasing trend in NLRP3 (**Figures 8F–G**).

## 4 Discussion

FZHFZY was a therapeutic medicinal plant formula used in clinical practice under the guidance of ancient empirical philosophy with the effect of enhancing the body resistance and dispelling dampness, easing the skin and relieving itching. A clinical retrospective study confirmed that FZHFZY significantly improved PASI and BSA scores in psoriasis patients, and its therapeutic effect was further enhanced when combined with antihistamines or other oral traditional Chinese medicines (15). FZHFZY treatment significantly inhibited the inflammation by modulating the expression of mediators, such as IL-23, IL-6, TNF-α, and IL-17A. FZHFZY was observed to inhibit the P38/Erk/NF-κB signaling pathways and Akt/mTORC1/S6K1 pathways (12), nevertheless, its precise mechanism remains undefined. In our study, we demonstrated through animal experiments that FZHFZY exhibited comparable efficacy to topical calcipotriol cream. On this basis, we used public databases combined with network pharmacology to predict potential targets of psoriasis, and further integrated metabolomic and transcriptomic analyses to explore the molecular pathways involved in FZHFZY treatment, providing valuable insights for further investigation of its underlying molecular mechanisms.

In this study, we delved deeper into the molecular mechanisms underpinning FZHFZY’s pharmacological effects using network pharmacology. An innovative approach was adopted for external use formulas by utilizing targets derived from skin diseases. Unlike current network pharmacology studies where disease targets for external formulas are directly sourced from databases, these targets were identified through analysis of the GEO database (16). Our approach predicted the potential herb-compound-target pathway interactions associated with psoriasis, providing new insights into the complex interplay between traditional Chinese medicine and modern molecular targets. Network pharmacology showed that these mechanisms may influence IL-17 signaling pathway, cytokine-cytokine receptor interaction and TNF signaling pathway. FZHFZY may have a therapeutic effect on psoriasis via the pathways of inflammation, proliferation, and metabolism.

NOD-like receptors (NLRs) have been found to play essential roles in processes such as inflammatory responses and the regulation of antigen presentation (17–19). Among these, extensive research has focused on the roles of inflammasomes formed by NLR family members (e.g., NLRP1 and NLRP3) and the AIM2-like receptor family member AIM2 in inflammatory skin diseases (20). Research has indicated that the inflammasome inhibitor (NLRP2/AIM-3-IN-21) exhibited significant therapeutic potential in psoriasis, and effectively suppressed the expression levels of NLRP3, IL-17A, and AIM2 in psoriatic skin lesion tissues (21). Additionally, topical application of Cornus officinalis seed extract inhibited the cleavage of the inflammasome activation marker caspase-1, alleviating psoriasis-like symptoms like desquamation, erythema, epidermal thickening (22). Silencing NLRP3 gene can inhibit the proliferation of psoriasis-like HaCaT cells, arrest cell cycle, inhibit the expressions of cell proliferation-related proteins and reduce levels of pro-inflammatory factors (23). The IL-23/Th17 signaling axis is recognized as the core pathway in the immunopathogenesis of psoriasis (24), where the cytokines IL-17 and IL-23 act synergistically to drive immune-inflammatory responses in skin tissues (25). Keratinocytes respond to NET-associated RNA (naRNA) with expression of psoriasis-related genes (IL-17) via TLR8-NLRP3 inflammasome-dependent pathway (26). Our study found that that FZHFZY effectively ameliorated psoriasiform dermatitis in IMQ-treated mice (PASI score), reduced lesion thickness, and decreased the expression levels of CD3 and Ki67. Through RNA-sequencing analysis, we investigated the gene expression profiles and functional changes in the IMQ-triggered psoriasis-like dermatitis mouse model following FZHFZY treatment. The top ranked NLR signaling pathway, IL-17 signaling pathway may contribute significantly to psoriasis skin lesions and pathways of FZHFZY treatment. Superior NLR signaling pathways were analyzed by comparing with psoriatic skin, uninvolved psoriatic skin and normal skin biopsies (27). MCODE analysis further identified six key genes associated with the NOD pathway, which could be pivotal in driving psoriatic inflammation. Notably, genes such as Nlrp3, Casp1, Casp4, IL-1β, IL-18, and Stat1 had high degree values, indicative of their significant position in the PPI network and potential targets for FZHFZY treatment of psoriasis. The critical genes were Nlrp3, IL-1β and Casp1. In summary, this study confirmed that the molecular mechanism of FZHFZY in treating psoriasis is associated with the regulation of the NLR and IL-17 signaling pathways, contributing to the further development of this formula.

A systematic review study has indicated that metabolites in the skin were considerably altered by psoriasis (28). Research has shown that the biochemical pathways of central metabolites used MetaboAnalyst (29), including folate biosynthesis and galactose metabolism, were remarkably changed in the cutaneous tissues of IMQ-induced psoriatic mice, and it also significantly changed histidine metabolism in IMQ-induced mouse skin tissues. FZHFZY improved the disorder of metabolites in the process of carbohydrate, purine and most amino acid catabolism. The metabolites were enriched in metabolism pathways, such as purine and nucleotide metabolisms. The analysis of purine metabolism was consistent with the findings of human studies (30). Research has shown that by quantifying the expression levels of GMP, AMP, and IMP purine monophosphate in lymphocytes, red blood cells, and epidermal tissues of healthy individuals and psoriatic patients, the terminal metabolites of xanthine, hypoxanthine, and uric acid metabolism are accumulating, and the activity of ADA and PNP in the psoriasis patients skin is increasing (31). Therefore, some researchers believe that purine nucleoside adenosine is the main regulatory factor of local tissue function when energy supply cannot meet the energy demand of cells (32).

In addition, we further conducted an integrated analysis of the differentially expressed genes identified in RNA-sequencing and differential metabolites. The pathway of purine metabolism analysis indicated that FZHFZY treated psoriatic skin lesion by regulating purine metabolism taken together, FZHFZY administration reversed changes in metabolites in several psoriasis-related metabolic pathways to treat psoriasis. Network analysis revealed that Adcy8 performs an essential role as a core node in the purine metabolism pathway. Its interaction with key molecules such as ATP and cAMP was indicative of its multifunctionality in signal transduction. Additionally, other nodes such as GMPR and xanthine oxidase were also identified. Heatmap analysis showed a significant negative correlation between Adcy8 and NLRP3, suggestive of a potential interaction and balance mechanism between the two in the purine metabolism pathway. Adcy8 is known to be responsible for the synthesis of cAMP, which is a crucial intracellular signaling molecule involved in various cellular physiological processes. Meanwhile, the NLRP3 inflammasome activation is intimately related to the pathological process of inflammatory diseases, and the increase in cAMP levels is believed to inhibit the activation of the NLRP3 inflammasome, exerting an anti-inflammatory effect.

Studies have shown that multiple immune cells are involved in the development and progression of psoriasis, with increasing attention being given to the important role of macrophages in psoriasis (33). Research has reported that during IMQ-induced psoriasis lesion progression, the damaged keratinocytes release extracellular vesicles carrying LRG1, which induce M1 macrophage polarization through TGFβ receptor 1. This upregulates the expression of inflammatory cytokines such as IL-1β and TNF-α, thereby promoting the development of psoriasis lesions (34). Studies have shown that cytokine-treated macrophages lead to clinical manifestations and histopathological alterations in psoriasiform dermatitis. Through body weight monitoring, skin shedding, erythema, hardness and thickness scoring, as well as skin pathology, it was verified that IL-23-activated macrophages exacerbated psoriasiform dermatitis in mice. Further validation by RT-PCR confirmed that IL-23 directly acted on macrophages, promoting the levels of IL-17A, IL-17F, IL-22, and IFN-γ, which may contribute to the severity of psoriasis in mice (35). Ubiquitin-specific protease 19 (USP19) serves as a molecular switch that reprograms NLRP3 from pro-inflammatory to anti-inflammatory activity (36). This was consistent with the findings of the current study, which demonstrated that FZHFZYF can suppress the secretion of inflammatory cytokines (such as IL-6, IL-23, etc.).

Although we have conducted preliminary research on the molecular mechanisms of FZHFZY in the treatment of psoriasis, our study still have some limitations. Due to the difficulty of obtaining skin samples from clinical patients, the number of psoriasis patients used to verify the skin expression levels of NLRP3 and IL-1β was relatively small. However, this conclusion has already been confirmed in previous studies. In addition, due to the limitations of mass spectrometry database matching, some active compounds in FZHFZY may not yet have been identified, warranting further in-depth investigation in the future.

## 5 Conclusion

Cumulatively, our results indicated that FZHFZY effectively attenuated psoriasis-like skin inflammation in IMQ-treated mice, as evidenced by pharmacodynamic indicators and immunohistochemistry. FZHFZY modulated key inflammatory pathways, including the NLR and IL-17 signaling pathways, and regulated metabolic processes such as carbohydrate, purine, and amino acid catabolism, as revealed by RNA-seq and metabolomic analyses. Integrative analysis of metabolome and transcriptome data indicated that the therapeutic effect of FZHFZY involved the regulation of purine and lipid metabolism, targeting critical genes in the NLR signaling pathway and modulating the skin immune microenvironment.

## Data Availability

The SRA records will be accessible using the following link after the indicated release date: https://www.ncbi.nlm.nih.gov/sra/PRJNA1366763.
