# Supplementary material for: Exploring therapeutic mechanism of Fuzhenghefuzhiyang Formula in psoriasis: inflammation and metabolism regulation
Source: Front Immunol. 2025 Nov 26;16:1690070. doi: 10.3389/fimmu.2025.1690070 (PMC12689303; doi:10.3389/fimmu.2025.1690070)
Supplement: Supplementary file 1 [file Presentation1.pdf]

Supplementary Material

1 Supplementary Figures and Tables

For more information on Supplementary Material and for details on the different file types accepted, please see here.

1.1 Supplementary Figure

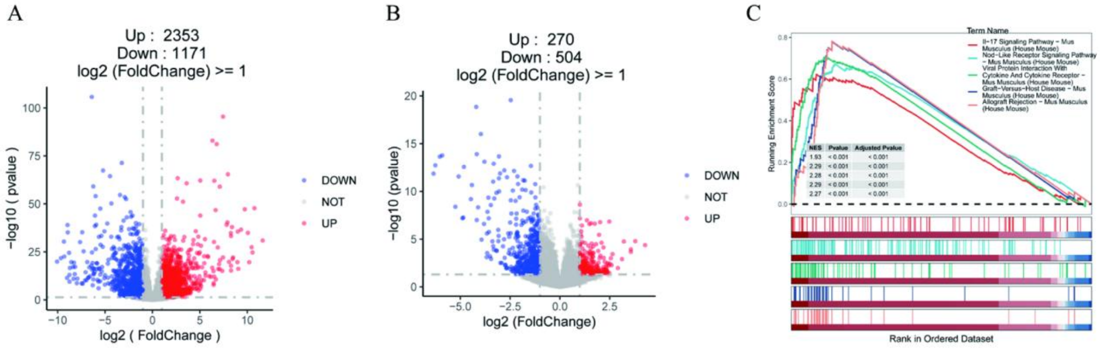

Figure S1 Transcriptome profiling. (A-B) the volcano map showed the distribution of DEGs. (C) the subsequent intersection analysis of these DEGs.

1.2 Supplementary Table

Supplemental Table 1. Analysis of the components in the FZHFZY.

NO.	Compound	tR/min	Molecular formula	Measured m/z	Expected Mass Spectra (m/z)	Ionization mode	Error（ppm）	MS/MS	Source	Ref.
1	Adenosine	1.42	$C_{10}H_{13}N_5O_4$	268.10458	268.10403	$[M+H]^+$	2.051	136.06165, 57.03426	*Rehmannia glutinosa* Libosch.	(Tsai *et al.*, 2010)
2	Decaffeoyl verbascoside	3.52	$C_{20}H_{30}O_{12}$	461.16794	461.16645	[M-H]-	3.231	315.10809, 297.09784, 161.04420, 135.04376	*Rehmannia glutinosa* Libosch.	(Wang *et al.*, 2020)
3	Chlorogenic acid	3.82	$C_{16}H_{18}O_9$	353.08542	353.08671	$[M-H]^-$	-3.653	191.05496, 173.04437	*Dictamnus dasycarpus* Turcz.	(Wang *et al.*, 2015)
4	Bergaptol	10.50	$C_{11}H_6O_4$	203.03345	203.03389	$[M+H]^+$	-2.167	147.04399, 119.04987	*Cnidium monnieri*（L.）Cuss.	(Lee *et al.*, 2016)
5	Xanthotoxol	10.67	$C_{11}H_6O_4$	203.03343	203.03389	$[M+H]^+$	-2.266	147.04387, 131.04903, 103.03930, 91.03939	*Cnidium monnieri*（L.）Cuss.	(Ngo *et al.*, 2017)
6	Jionoside B	10.91	$C_{37}H_{50}O_{20}$	813.28480	813.28227	[M-H]-	3.111	637.23480, 491.17731, 175.03891, 151.00237	*Rehmannia glutinosa* Libosch.	(Han *et al.*, 2022)
7	Astilbin	10.94	$C_{21}H_{22}O_{11}$	449.10742	449.10893	[M-H]-	-3.362	303.05060, 285.04041, 151.00243	*Dictamnus dasycarpus* Turcz.	(Farag *et al.*, 2015)
8	Isoverbascoside	11.08	$C_{29}H_{36}O_{15}$	623.19733	623.19814	[M-H]-	-1.300	461.16647, 315.10806, 161.02316, 135.04373	*Rehmannia glutinosa* Libosch.	(Ling *et al.*, 2022)
9	Forsythoside A	11.18	$C_{29}H_{36}O_{15}$	623.19733	623.19814	[M-H]-	-1.300	477.13861, 315.14737, 135.04373	*Rehmannia glutinosa* Libosch.	(Wang *et al.*, 2018)

10	Jionoside D	12.06	$C_{30}H_{38}O_{15}$	637.21429	637.21379	[M-H]-	0.785	475.18240, 461.16638, 315.10837, 193.04959, 161.02319, 135.04375	*Rehmannia glutinosa* Libosch.	(Han et al., 2022)
11	Isomaculosidine	14.85	$C_{14}H_{13}NO_4$	260.09164	260.09173	$[M+H]^+$	-0.346	245.06815, 217.08466	*Dictamnus dasycarpus* Turcz.	(Chang *et al.*, 2021)
12	Naringenin	15.00	$C_{15}H_{12}O_5$	271.06128	271.0601	[M-H]-	4.353	151.00241, 119.04877	*Smilax glabra* Roxb.	(Wen *et al.*, 2012)
13	Osthole	16.37	$C_{15}H_{16}O_3$	245.11713	245.11722	$[M+H]^+$	-0.367	189.05434, 161.05984, 159.04390, 131.049865	*Cnidium monnieri*（L.）Cuss.	(Song *et al.*, 2015)
14	Rutaevine	16.73	$C_{26}H_{30}O_9$	485.17969	485.18061	[M-H]-	-1.896	423.17657, 397.20285, 383.14627	*Dictamnus dasycarpus* Turcz.	(Zhou *et al.*, 2010)
15	Skimmianine	16.98	$C_{14}H_{13}NO_4$	260.09174	260.09173	$[M+H]^+$	0.038	245.06812, 227.05699, 199.11214	*Dictamnus dasycarpus* Turcz.	(Chang *et al.*, 2021)
16	Dehydroxypeucedanin hydrate	17.01	$C_{15}H_{14}O_3$	243.10185	243.10157	$[M+H]^+$	1.152	189.05432, 159.04407, 131.04886	*Cnidium monnieri*（L.）Cuss.	(Li and Chan, 2013)
17	Bergapten	17.23	$C_{12}H_8O_4$	217.04927	217.04954	$[M+H]^+$	-1.244	202.02571, 174.06320	*Cnidium monnieri*（L.）Cuss.	(Seo, 2023)
18	Xanthotoxin	17.42	$C_{12}H_8O_4$	217.04915	217.04954	$[M+H]^+$	-1.797	202.02568, 174.06400, 161.05948, 131.08626	*Dictamnus dasycarpus* Turcz.	(Song *et al.*, 2015)
19	Limonin	17.49	$C_{26}H_{30}O_8$	471.20029	471.20134	$[M+H]^+$	-2.228	427.20728, 425.19492, 161.05959	*Dictamnus dasycarpus* Turcz.	(Zhou *et al.*, 2010, Wang *et al.*, 2013)
20	Obacunone	17.53	$C_{26}H_{30}O_7$	455.20435	455.20643	$[M+H]^+$	-4.569	437.19485, 409.19928, 391.18906, 359.12640, 331.13184, 161.05948	*Dictamnus dasycarpus* Turcz.	(Wang *et al.*, 2013)
21	Obacunonic acid	18.19	$C_{26}H_{32}O_8$	473.21716	473.21699	$[M+H]^+$	0.359	455.20441, 437.19156, 427.21106, 411.21362,	*Dictamnus dasycarpus* Turcz.	(Liu *et al.*, 2015)

								409.20020		
22	Isopimpinellin	18.74	$C_{13}H_{10}O_5$	247.06010	247.0601	$[M+H]^+$	0.000	232.03656, 217.01280, 203.10632, 189.07359, 161.09688	*Dictamnus dasycarpus* Turcz.	(Song *et al.*, 2015)
23	Dictamnine	18.78	$C_{12}H_9NO_2$	200.07024	200.07061	$[M+H]^+$	-1.849	185.04668, 129.07027	*Dictamnus dasycarpus* Turcz.	(Chang *et al.*, 2021, Wang et al., 2013)
24	γ-Fagarine	19.14	$C_{13}H_{11}NO_3$	230.08189	230.08117	$[M+H]^+$	3.129	215.05646, 200.04648	*Dictamnus dasycarpus* Turcz.	(Chang *et al.*, 2021)
25	Dasycarpanine	19.54	$C_{17}H_{21}NO_4$	304.15427	304.15442	$[M+H]^+$	-0.493	286.18060, 201.08914	*Dictamnus dasycarpus* Turcz.	(Wang *et al.*, 2013)
26	Imperatorin	19.73	$C_{16}H_{14}O_4$	271.09573	271.09649	$[M+H]^+$	-2.803	203.03346, 175.11137, 147.08096, 131.08556, 67.07067	*Rehmannia glutinosa* Libosch.	(Ngo *et al.*, 2017)
27	Isopteleine	19.84	$C_{13}H_{11}NO_3$	230.08217	230.08117	$[M+H]^+$	4.346	215.05702, 200.03313, 187.11139, 172.95210	*Dictamnus dasycarpus* Turcz.	(Chang *et al.*, 2021)
28	Fraxinellone	20.56	$C_{14}H_{16}O_3$	233.11682	233.11722	$[M+H]^+$	-1.716	215.106268, 187.11147, 159.11679	*Dictamnus dasycarpus* Turcz.	(Wang *et al.*, 2013)

References

CHANG, K., GAO, P., LU, Y., TU, P., JIANG, Y. & GUO, X. (2021), "Identification and characterization of quinoline alkaloids from the root bark of Dictamnus dasycarpus and their metabolites in rat plasma, urine and feces by UPLC/Qtrap-MS and UPLC/Q-TOF-MS", *J Pharm Biomed Anal,* Vol. 204114229.

FARAG, M. A., SAKNA, S. T., EL-FIKY, N. M., SHABANA, M. M. & WESSJOHANN, L. A. (2015), "Phytochemical, antioxidant and antidiabetic evaluation of eight Bauhinia L. species from Egypt using UHPLC-PDA-qTOF-MS and chemometrics", *Phytochemistry,* Vol. 11941-50.

HAN, D., YUE, Z., LI, H., LIU, G., CAI, B. & TIAN, P. (2022), "[Anti-depressant components and mechanism of Rehmanniae Radix based on UPLC-Q-Orbitrap HRMS and network pharmacology]", *Zhongguo Zhong Yao Za Zhi,* Vol. 47 No. 4, pp. 1051-1063.

LEE, S. G., KIM, K., VANCE, T. M., PERKINS, C., PROVATAS, A., WU, S., QURESHI, A., CHO, E. & CHUN, O. K. (2016), "Development of a comprehensive analytical method for furanocoumarins in grapefruit and their metabolites in plasma and urine using UPLC-MS/MS: a preliminary study", *Int J Food Sci Nutr,* Vol. 67 No. 8, pp. 881-7.

LI, J. & CHAN, W. (2013), "Investigation of the biotransformation of osthole by liquid chromatography/tandem mass spectrometry", *J Pharm Biomed Anal,* Vol. 74156-61.

LING, Z., ZENG, R., ZHOU, X., CHEN, F., FAN, Q., SUN, D., CHEN, X., WEI, M., WU, R. & LUO, W. (2022), "Component analysis using UPLC-Q-Exactive Orbitrap-HRMS and quality control of Kudingcha (Ligustrum robustum (Roxb.) Blume)", *Food Res Int,* Vol. 162 No. Pt A, pp. 111937.

LIU, X., ZHANG, F., GAO, S., JIANG, B. & CHEN, W. (2015), "Metabolite profiling of Zi-Shen pill in rat biological specimens by UPLC-Q-TOF/MS", *Chin J Nat Med,* Vol. 13 No. 2, pp. 145-60.

NGO, L., TRAN, P., HAM, S., CHO, J., CHO, H. & LEE, Y. (2017), "Simultaneous determination of imperatorin and its metabolite xanthotoxol in rat plasma and urine by LC-MS/MS and its application to pharmacokinetic studies", *J Chromatogr B Analyt Technol Biomed Life Sci,* Vol. 1044-104530-38.

SEO, C. (2023), "Simultaneous Analysis of Bergapten and Schinifoline in Zanthoxylum schinifolium Seeds Using HPLC and UPLC-MS/MS Systems", *Foods,* Vol. 12 No. 7, pp.

SONG, G., ZHOU, L., SHENG, N., ZHANG, X., XU, Y., ZHANG, L. & LI, X. (2015), "Simultaneous quantification of 16 bioactive constituents in Common cnidium fruit by liquid chromatography-electrospray ionization-mass spectrometry", *J Pharm Biomed Anal,* Vol. 107304-10.

TSAI, Y., LIN, L. & TSAI, T. (2010), "Pharmacokinetics of adenosine and cordycepin, a bioactive constituent of Cordyceps sinensis in rat", *J Agric Food Chem,* Vol. 58 No. 8, pp. 4638-43.

WANG, F., CAO, G., LI, Y., XU, L., WANG, Z., LIU, Y., LU, J. & ZHANG, J. (2018), "Characterization of forsythoside A metabolites in rats by a combination of UHPLC-LTQ-Orbitrap mass spectrometer with multiple data processing techniques", *Biomed Chromatogr,* Vol. 32 No. 5, pp. e4164.

WANG, H., SUN, H., ZHANG, A., LI, Y., WANG, L., SHI, H., DIZOU, X. L. & WANG, X. (2013), "Rapid identification and comparative analysis of the chemical constituents and metabolites of Phellodendri amurensis cortex and Zhibai dihuang pill by ultra-performance liquid chromatography with quadrupole TOF-MS", *J Sep Sci,* Vol. 36 No. 24, pp. 3874-82.

WANG, P., SUN, J., GAO, E., ZHAO, Y., QU, W. & YU, Z. (2013), "Simultaneous determination of limonin, dictamnine, obacunone and fraxinellone in rat plasma by a validated UHPLC-MS/MS and its application to a pharmacokinetic study after oral administration of Cortex Dictamni extract", *J Chromatogr B Analyt Technol Biomed Life Sci,* Vol. 92844-51.

WANG, X., WU, C., XU, M., CHENG, C., LIU, Y. & DI, X. (2020), "Optimisation for simultaneous determination of iridoid glycosides and oligosaccharides in Radix Rehmannia by microwave assisted extraction and HILIC-UHPLC-TQ-MS/MS", *Phytochem Anal,* Vol. 31 No. 3, pp. 340-348.

WANG, Y., WEN, J., ZHENG, W., ZHAO, L., FU, X., WANG, Z., XIONG, Z., LI, F. & XIAO, W. (2015), "Simultaneous determination of neochlorogenic acid, chlorogenic acid, cryptochlorogenic acid and geniposide in rat plasma by UPLC-MS/MS and its application to a pharmacokinetic study after administration of Reduning injection", *Biomed Chromatogr,* Vol. 29 No. 1, pp. 68-74.

WEN, J., QIAO, Y., YANG, J., LIU, X., SONG, Y., LIU, Z. & LI, F. (2012), "UPLC-MS/MS determination of paeoniflorin, naringin, naringenin and glycyrrhetinic acid in rat plasma and its application to a pharmacokinetic study after oral administration of Si-Ni-San decoction", *J Pharm Biomed Anal,* Vol. 66271-7.

ZHOU, X., ZHAO, Y., LEI, P., CAI, Z. & LIU, H. (2010), "Chromatographic fingerprint study on Evodia rutaecarpa (Juss.) Benth by HPLC/DAD/ESI-MS(n) technique", *J Sep Sci,* Vol. 33 No. 15, pp. 2258-65.
